# Surveillance of Clostridium difficile infections in Finnish acute care hospitals, 2008-2014: trends, diagnostics and control measures

**DOI:** 10.1186/2047-2994-4-S1-P22

**Published:** 2015-06-16

**Authors:** DK Arifulla, S Mentula, SM Kotila, J Ollgren, T Möttönen, O Lyytikäinen

**Affiliations:** 1 National Institute for Health and Welfare, Helsinki, Finland

## Introduction

Enhanced hospital-based surveillance of *Clostridium difficile* infections (CDI) started after the first detection of PCR ribotype 027 in October 2007 as part of Finnish Hospital Infection Program.

## Objectives

The aim was to evaluate trends in CDI rates in relation to diagnostic methods and control measures used in participating hospitals.

## Methods

Prospective laboratory-based surveillance was conducted using case definitions of the European Centre for Disease Prevention and Control. Participating hospitals were given 1-day training session on surveillance methods and control measures based on European guidelines. As a feedback the hospitals were anonymously ranked by the overall and nosocomial incidence rates of CDI, and prevalence of CDI among admitted patients. Laboratories serving the hospitals were asked to send isolates from severe cases and persistent outbreaks to the national reference laboratory for genotyping. A survey on diagnostic methods and infection control measures was performed in March 2015.

## Results

A total of 5753 CDI cases were identified in 18 hospitals; 4165 (72%) were nosocomial. There were significant decreasing trends (p<.05) in overall rate, nosocomial rate and prevalence at admission which decreased from 0.78/1000 patient-days to 0.40, 0.53/1000 patient-days to 0.31 and 0.72/1000 admissions to 0.23, respectively. All but one hospital had access to PCR; 10 used it as a primary detection method, others after culture or GDH test. Chlorine was used as environmental disinfecting agent in 12 hospitals and H2O2 in 6. Hand hygiene included both washing and disinfection in all but one hospital. Of all isolates sent to reference laboratory, the proportion of isolates from persistent outbreaks decreased from 47% to 13%.

## Conclusion

Despite the increased usage of more sensitive methods for detecting CDI, there was a reduction in CDI rates during 2008-2014, which is likely related to increased awareness of CDI and improved control measures. When ranking hospitals the diagnostic activity should also be evaluated.

## Disclosure of interest

None declared.

